# Differences in access and utilisation of mental health services in the perinatal period for women from ethnic minorities—a population-based study

**DOI:** 10.1186/s12916-020-01711-w

**Published:** 2020-09-11

**Authors:** Jelena Jankovic, Jake Parsons, Nikolina Jovanović, Giles Berrisford, Alex Copello, Qulsom Fazil, Stefan Priebe

**Affiliations:** 1 Perinatal Mental Health Service, Birmingham and Solihull Mental Health NHS Foundation Trust, The Barberry, 25 Vincent Drive, Birmingham, B15 2FG UK; 2 The Strategy Unit (Hosted by Midlands and Lancashire Commissioning Support Unit), 5th Floor Kingston House, 438-450 High Street, West Bromwich, B70 9LP UK; 3 Unit for Social and Community Psychiatry (WHO Collaborating Centre for Mental Health Services Development), Newham Centre for Mental Health, Bart’s and London School of Medicine and Dentistry, Queen Mary University of London, London, E13 8SP UK; 4 School of Psychology, University of Birmingham, 52 Pritchatts Road, Birmingham, B15 2SA UK; 5 Research and Innovation, Birmingham and Solihull Mental Health Foundation Trust, National Center for Mental Health, The Barberry 25 Vincent Drive, Edgbaston, Birmingham, B15 2FG UK; 6 Institute of Applied Research, College of Medical and Dental Sciences, University of Birmingham, Edgbaston, Birmingham, B15 2TT UK

**Keywords:** Ethnicity, Perinatal, Mental health, Psychiatry, Access

## Abstract

**Background:**

Barriers to accessing mental health care during pregnancy and the first postnatal year (perinatal period) seem to be greater for ethnic minority women; however, there is no reliable large-scale data about their actual use of mental health services during this period. Our study aims to explore access rates to secondary mental health services, including involuntary admissions to psychiatric inpatient care and patterns of engagement for ethnic minority women aged 18+ who gave birth in 2017 in England, UK.

**Methods:**

Two datasets from the National Commissioning Data Repository, the Acute Inpatient Dataset and Mental Health Services Dataset, were linked. Datasets covering the full perinatal period for each woman were included. Rates were standardised by age and deprivation.

**Results:**

Out of 615,092 women who gave birth in England in 2017, 22,073 (3.5%) started a contact with mental health services during the perinatal period. In total, 713 (3.2%) were admitted to inpatient care, and 282 (39.5%) involuntarily. Ethnicity data was available for 98% of the sample. Black African, Asian and White Other women had significantly lower access to community mental health services and higher percentages of involuntary admissions than White British women. Black African, Asian and White Other women had a higher number of attended community contacts and fewer non-attendances/cancellations of appointments than White British women.

**Conclusion:**

Access to mental health services during the perinatal period varies significantly between women from different ethnic groups. Access to community mental health services should be facilitated for Black African, Asian and White Other women during the perinatal period, which may reduce rates of involuntary hospital admissions for these groups. The pattern of engagement with community services for women from these ethnicities indicates that access appears to be a problem rather than utilisation.

## Background

Mental illness is common during pregnancy and first postnatal year (perinatal period), and up to 20% of women experience a wide range of mental health conditions [1, 2]. In this paper, we define period of pregnancy and first postnatal year as the perinatal period in line with the definition by NHS England [1]. Women with perinatal mental illness can face various barriers to accessing mental health services at individual and organisational levels [3, 4]. These barriers were identified at four levels: individual (e.g, stigma, poor awareness), organisational (e.g. resource inadequacies, service fragmentation), sociocultural (e.g. language/cultural barriers) and structural (e.g. unclear policy) levels [4]. As a consequence of these barriers, mental illness during the perinatal period frequently remains untreated. This can have a significant negative impact on the health of the mother and the health of their children, on her partner and the wider family and on the society as a whole [5].

Some findings suggest that for women from ethnic minorities, barriers to accessing mental health care during the perinatal period exists to even a greater extent [4, 6]. The evidence on ethnic minority women’s experiences of perinatal mental health conditions and utilisation of services suggests that some women were not aware of the support available and that access to services was influenced by a number of individual and organisational issues [6]. Evidence from a large nationally representative sample of mothers reported that Black and minority ethnic as well as migrant women were less likely to report treatment for anxiety/depression [7]. Another study from the United States (US) showed that pregnant women with mental health/substance use disorders from ethnic minority groups had significantly lower odds of receiving mental health treatment compared to White women [8].

In addition to the barriers potentially faced by all women, women from ethnic minorities may also have to deal with language problems, different cultural explanatory models of mental illness and expectations about appropriate help, and a lack of understanding of the health care system. In one study, a diagnosis of common mental disorders during the maternal period was twice as likely to be missed in minority ethnic women (maternal period was defined as 6 months prior to conception, through pregnancy and 1 year after delivery) [9] In a United Kingdom (UK) national survey, non-White women were less likely to be asked about their mental health, to be offered treatment or to receive support in the postnatal period [10]. In England, in the population of women who give birth, the proportion of women from ethnic minorities is higher than from general population [11, 12], and perinatal mental health services should provide equitable access and meet the needs of ethnic minority women and their families. However, there are no reliable large-scale data about the actual use of mental health services by women from ethnic minorities during the perinatal period. Such data are essential to inform current policies which envisage an unprecedented expansion of specialist perinatal mental health services as part of the Five Year Forward View for Mental Health in England [13] and the National Health Service (NHS) Long Term Plan [14].

This study aims to explore access rates to secondary mental health services and patterns of engagement with these services for women from ethnic minority groups in the perinatal period in England by the following:

1. Exploring access rates to community mental health services, rates of inpatient psychiatric hospital admissions and rates of involuntary inpatient psychiatric hospital admissions
2. Exploring whether a higher density of ethnic minority populations is linked to lower access rates
3. Exploring the number of contacts with community mental health services, the number of cancellations and the number of non-attendances

## Methods

The primary source of data for this work is the National Commissioning Data Repository (NCDR). This is a national dataset held by NHS England containing up-to-date extracts of secondary uses service (SUS) health care data. Two key datasets within the NCDR used are (1) acute inpatient dataset—to identify women in England who had a hospital birth episode; (2) Mental Health Services Dataset (MHSDS)—to identify women in England who had contact with any type of secondary mental health services, including admissions to psychiatric inpatient care. The two datasets are linked using the unique pseudonymised NHS number for each patient which is a required field in both datasets. The data completeness of these fields is good with 96.2% of birth episodes and 98.8% of records within the MHSDS having a valid pseudonymised NHS number.

The acute inpatient dataset was used to identify women who had given birth in an NHS hospital during the calendar year 2017. Birth episodes were identified using the Health Resource Group (HRG) tariff code field within the NCDR inpatient dataset. HRG codes are used for payments to providers. Birth episodes can be identified by their HRG code, for example, a hospital episode with code NZ50C indicates a planned C-section without complications. All hospital episodes with one of a number of HRG codes that indicate the delivery of a baby were extracted from this database. The quality of birth records within this dataset is considered to be good as it corresponds closely with the Office for National Statistics data on recorded births (see the “Results” section for more details).

The birth episode dataset was linked to the MHSDS using the pseudonymised NHS number available within both the acute inpatient dataset and the Master Patient Index table within the MHSDS. This allowed us to establish the unique mental health person identifier which is the primary patient identifier used to link tables within the MHSDS database related to different activity types (such as contacts and admissions). Using this identifier, all care contacts recorded within the MHSDS between 1 January 2016 and 31 December 2018 for women who had a birth episode were extracted from table MHS201 of the MHSDS (contacts extracted included cancelled or not attended contacts as these are indicative of access to support even when not attended). All mental health inpatient spells recorded within the MHSDS between 1 Jan 2016 and 31 December 2018 for women who had a birth episode were extracted from table MHS501 of the MHSDS. Including service use data up to the end of 2018, we used the most recent available data for full calendar years.

Using these extracts, three subgroups of women who had a recorded birth episode in 2017 were identified for the purpose of this analysis. These three groups were identified as follows: (1) Women aged 18+ with a birth episode in 2017 who had at least one community mental health service contact (such as contact with community mental health teams, crisis resolution and home treatment teams, perinatal mental health teams) recorded and whose first recorded contact (within the extracted dataset) was during their perinatal period (defined as birth date minus recorded gestation length or, where gestation length was not recorded, minus 39 weeks to 12 months after the birth date). We conducted the analysis only with women who started their contact with mental health services in the perinatal period and excluded women who had a contact recorded within the data extract that occurred prior to conception and as such had already accessed and were engaged with mental health services prior to the perinatal period. Some of those women may have been in contact with mental health services in the past but were not in contact at the time of conception. (2) Women aged 18+ who had at least one admission to psychiatric inpatient care within their perinatal period and (as for group 1) whose first recorded contact with mental health services was during their perinatal period. (3) Women aged 18+ whose first admission to psychiatric inpatient care during the perinatal period was involuntary under the Mental Health Act 1983. Women under 18 were excluded on the basis that access to and the nature of service provision for under 18 differs markedly from adult service provision as such it was felt their exclusion would help to minimise potential issues relating to differences in adult and child provision.

The ethnicity of women in the full dataset was taken from the “Ethnic Group” field within the acute inpatient dataset where this was completed. Where the ethnicity was missing from the acute inpatient record and the woman also had a record in the NCDR MHSDS Master Patient Index (MPI) table, then the ethnicity was taken from the most recent record in the MPI table. Patient ethnicity within the fields in both datasets is recorded using the classifications developed for the 2001 UK Census of ethnic groups. There are 16 ethnic categories that are grouped into 5 broad ethnic groups—White, Asian, Black, Mixed and Other [11, 15].

Rates of access to community mental health services within the perinatal period were calculated for women for each ethnic category. Contact rates were calculated as follows: the numerator was the number of women aged 18+ with a birth episode in 2017 who had their first contact with mental health services within their perinatal period; the number of birth episodes in 2017 was the denominator.

Rates of admissions to inpatient care during the perinatal period were calculated as follows: the numerator was the number of women aged 18+ with a birth episode in 2017 who had one or more psychiatric inpatient admissions during their perinatal period and where their first contact was also within their perinatal period; again, the number of birth episodes in 2017 was the denominator.

The rate of women with involuntary admissions, i.e. who were formally detained under the Mental Health Act 1983 during their first inpatient episode, was calculated as follows: the denominator was the number of women aged 18+ with a birth episode in 2017 who had one or more psychiatric inpatient admissions during their perinatal period and where their first contact was also within their perinatal period; the numerator was the number of those in the denominator who also had a Mental Health Act legal status classification starting on, during or up to 7 days prior to their first admission.

In the calculation of all these rates, both the numerator and denominator missing ethnicities were assumed to be missing at random and as such, they were inflated based on adding the number of missing ethnicities in proportion to known ethnicities.

As the literature suggests higher rates of mental health problems in younger pregnant women [16] and more deprived populations [17], simple crude contact rates could produce misleading results since different access rates might reflect different levels of need in different age and/or deprivation groups. To take account of the age and deprivation distributions in women from different ethnic groups, both crude and directly age and deprivation-standardised contact rates were calculated in all analyses except for involuntary admissions as the numbers were too small to be standardised by age and/or deprivation. Direct standardisation takes the age and deprivation-specific rates for each (ethnic/deprivation) group and applies them to the same standard population. In this case, the age and deprivation-specific rates were calculated using 5-year age bands and the index of multiple deprivation (IMD) decile. Data about age and deprivation distribution is included as Supplementary material.

In this paper, we define (1) “access to community mental health services” as having at least one recorded contact (whether attended or not), (2) “access to inpatient care” as an admission to a psychiatric hospital and (3) “utilisation of mental health services” as the number of attended contacts, non-attendances and cancellations in community mental health services.

Some evidence [18] suggests that levels of mental health problems may be lower in ethnic minority groups living in areas with a higher ethnic minority population density, as has been shown for rates of suicide. We therefore explored whether a higher density of ethnic minority populations was linked to lower access rates. We used the percentage of births for Black, Asian and White Other groups as a proxy for population density within geographical areas defined as related to individual clinical commissioning groups (CCG). Charts were produced illustrating the association of ethnic minority density with the contact rates of women with community mental health services. Pearson’s coefficient of determination (*r*^2^) values were calculated to establish the strength of the associations.

The study used pseudonymised data, and therefore, the ethics approval was not required and included patients did not provide informed consent.

In England, patients are usually referred to secondary mental health services via primary care (general practitioners, health visitors, midwives, etc.); however, for urgent referrals, there is an additional pathway (usually via emergency services to crisis and home treatment teams).

## Results

### Study sample

**Fig. 1:**
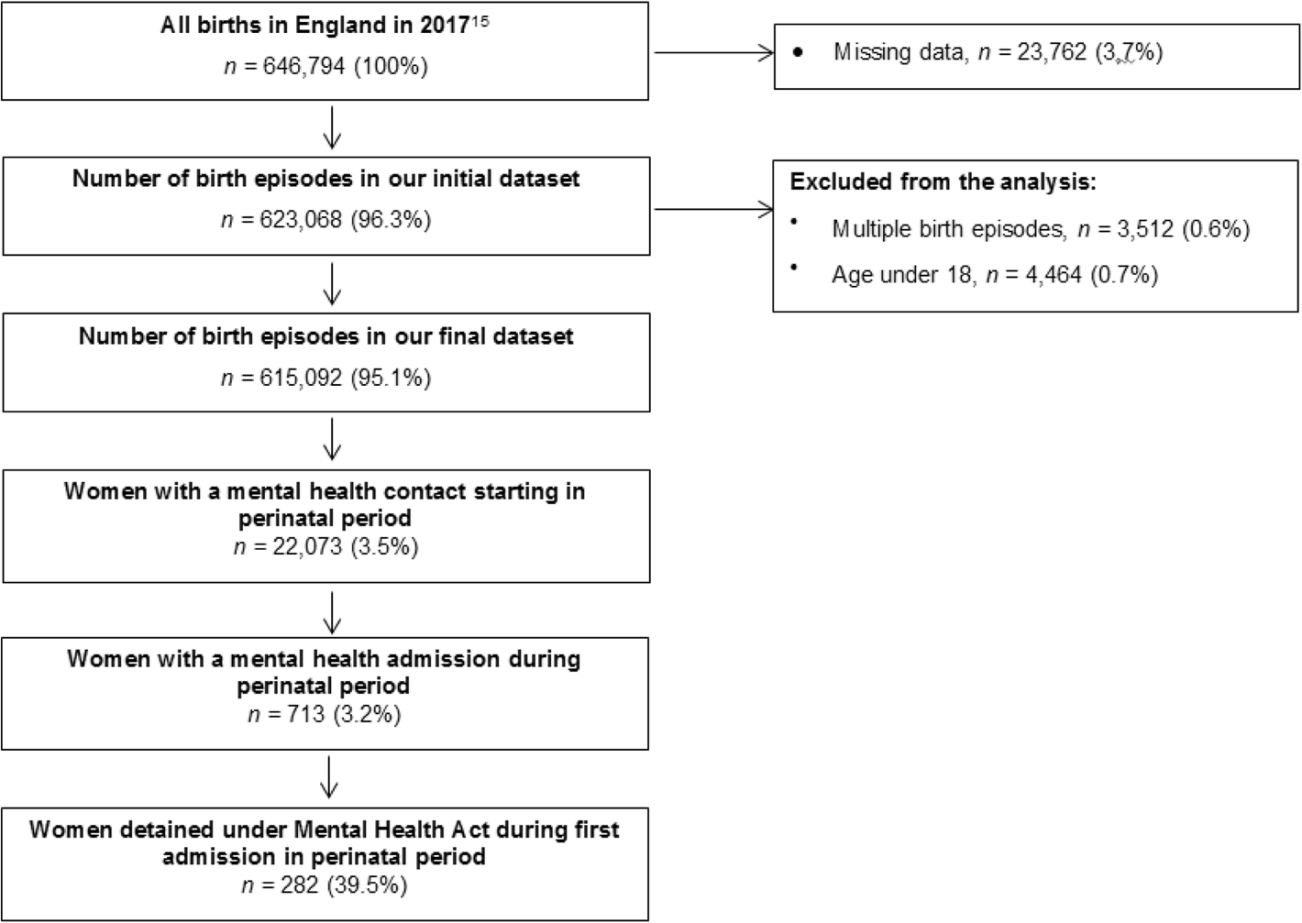
Study sample

The initial dataset comprised 623,068 birth episodes which is 96.3% of all births in England, UK, in 2017 (the number of births in England in 2017 was 646,794 according to the Office for National Statistics) [19]. The small discrepancy is likely to reflect women who gave birth at private hospitals or at home as these are not included in the acute inpatient dataset. In addition, the ONS figure is a count of registered births whereas the acute inpatient data counts birth episodes (the birth of twins is counted as a single birth episode as it relates to the mother and not the baby). There are around 10,000 births of twins, triplets or more each year. A small number of multiple birth episodes (3512; 0.6%) relating to women who had more than one birth episode during the period were excluded from the analysis due to the difficulty in linking the use of mental health services to specific births. Restricting the analysis to women who were 18 or more years of age resulted in a final birth episode dataset of 615,092 (95.1%) birth episodes. Out of those, 29,814 women had one or more recorded contacts with mental health services in the perinatal period. For 22,073, it was the first contact with mental health services. Thus, they accessed mental health services within the perinatal period as opposed to having already been known to them when they became pregnant. Out of these 22,073 women, 713 were admitted to psychiatric inpatient care during the perinatal period, and 282 of them involuntarily. Please see Fig. 1.

### Ethnicity

The ethnicity for 548,689 (89.2%) of the 615,092 women in the full dataset was recorded in the “Ethnic Group” field within the acute inpatient dataset. Using all available data in identified datasets, missing ethnicity data of women with contacts with mental health services were reduced to around 2% and amongst women with admissions to psychiatric inpatient care to only 0.3%. The ethnicity of all women who gave birth in 2017 and are recorded in the dataset is presented in Table 1.

**Table 1:** Ethnicity of women in the initial dataset and in subgroups of women in receipt of mental health support

	Ethnicity	All 18+, birth episodes, ***n*** (%)	A) Women with a mental health contact starting in the perinatal period, ***n*** (%)	B) Women with a mental health admission during the perinatal period with no contacts before the perinatal period, ***n*** (%)
**A**	**White British**	355,719 (57.8)	16,552 (75.0)	442 (62.0)
**B**	**Irish**	3028 (0.5)	111 (0.5)	**
**C**	**Other White backgrounds**	64,177 (10.4)	1493 (6.8)	67 (9.4)
**D**	**White and Black Caribbean**	3126 (0.5)	200 (0.9)	7 (1.0)
**E**	**White and Black African**	1453 (0.2)	64 (0.3)	**
**F**	**White and Asian**	1746 (0.3)	58 (0.3)	**
**G**	**Other mixed backgrounds**	3978 (0.6)	204 (0.9)	**
**H**	**Indian**	17,960 (2.9)	312 (1.4)	9 (1.3)
**J**	**Pakistani**	24,132 (3.9)	545 (2.5)	39 (5.5)
**K**	**Bangladeshi**	8492 (1.4)	219 (1.0)	19 (2.7)
**L**	**Other Asian backgrounds**	13,071 (2.1)	318 (1.4)	17 (2.4)
**M**	**Caribbean**	4883 (0.8)	259 (1.2)	12 (1.7)
**N**	**African**	18,009 (2.9)	428 (1.9)	39 (5.5)
**P**	**Any other Black backgrounds**	4130 (0.7)	192 (0.9)	13 (1.8)
**R**	**Chinese**	3822 (0.6)	79 (0.4)	**
**S**	**Other ethnic groups**	20,963 (3.4)	603 (2.7)	34 (4.8)
**NK**	**Not known**	66,403 (10.8)	436 (2.0)	**
	**Total**	615,092 (100)	22,073 (3.5)	713 (3.2)

The table shows the size, ethnic structure (including missing ethnicities) of the 2017 birth episode dataset and the subgroups considered within this analysis. A) Women with one or more mental health contacts during their perinatal period (PNP) where the first contact is within PNP. B) Women with one or more mental health inpatient admissions during the PNP where the first contact is also within PNP. Numbers and percentages for each subgroup are provided

**Numbers are suppressed due to small numbers < 5

It shows that in the initial dataset, 355,719 (57.8%) women were White British with White Other being the second biggest ethnic category (*n* = 64,177, 10.4%). From the Asian subgroup, Pakistani women were the biggest group (*n* = 24,132, 3.9%) followed by Indian (*n* = 17,960, 2.9%) whilst within the Black group, Black African women were the largest group (*n* = 18,009, 2.9%). Table 1 also shows the ethnicity of women who received mental health support during the perinatal period.

### Access to community mental health services

Numbers and rates of contacts with community mental health services are shown per 1000 births by ethnicity (crude and standardised by age and deprivation) in Table 2. We found that White Other women, Asian women (all subgroups including the mixed group White/Asian), Black African, Chinese and Other group women had significantly lower access to community mental health services than White British women. The mixed group White/Black Caribbean had statistically significantly higher access than White British women. Women of Black Caribbean ethnicity had higher rates of contact with community mental health services than White British women; however, the difference was not statistically significant.

**Table 2:** Numbers and rates of community contact with mental health services per 1000 births by ethnicity (crude and standardised by age and deprivation)

	Ethnicity	All 18+, birth episodes, ***n***	Women with a mental health contact, ***n***	Crude rate (per 1000)			Standardised rate (per 1000)		
				Rate	95% LCL	95% UCL	Rate	LCL	UCL
**A**	**White British**	398,769	16,886	42.3	41.7	43.0	42.4	41.8	43.1
**B**	**Irish**	3394	113	33.4*	27.5	40.1	38.6	31.1	47.3
**C**	**Other White backgrounds**	71,944	1523	21.2*	20.1	22.3	21.6*	20.6	22.8
**D**	**White and Black Caribbean**	3504	204	58.2*	50.5	66.8	51.7*	43.8	60.6
**E**	**White and Black African**	1629	65	40.1	31.0	51.1	45.1	33.9	58.7
**F**	**White and Asian**	1957	59	30.2*	23.0	39.0	31.2*	23.5	40.6
**G**	**Other mixed backgrounds**	4459	208	46.7	40.5	53.5	45.1	39.0	51.8
**H**	**Indian**	20,134	318	15.8*	14.1	17.6	17.3*	14.8	20.1
**J**	**Pakistani**	27,052	556	20.6*	18.9	22.3	19.4*	17.3	21.7
**K**	**Bangladeshi**	9520	223	23.5*	20.5	26.8	22.2*	18.1	26.9
**L**	**Other Asian backgrounds**	14,653	324	22.1*	19.8	24.7	22.0*	19.4	24.8
**M**	**Caribbean**	5474	264	48.3	42.6	54.5	43.9	37.8	50.7
**N**	**African**	20,188	437	21.6*	19.6	23.8	25.2*	21.9	28.7
**P**	**Any other Black backgrounds**	4630	196	42.3	36.6	48.7	42.5	35.2	50.9
**R**	**Chinese**	4285	81	18.8*	14.9	23.4	23.3*	16.5	31.8
**S**	**Other ethnic groups**	23,500	615	26.2*	24.1	28.3	25.7*	23.6	28.0
	**Total**	615,092	22,072						

The table shows the number of women with one or more mental health contacts during the perinatal period (PNP) where the first contact is within PNP and the total number of birth episodes in 2017 (events with missing ethnicities are included; assumed to be missing at random and are apportioned across all ethnicities in proportion to known ethnicities)

Crude and directly age-standardised rates per 1000 birth episodes are shown along with corresponding 95% confidence intervals

*LCL* lower confidence limit, *UCL* upper confidence limit

*Rate is significantly different (*p* < 0.05) from the White British group

### Access to inpatient mental health services

The rates of psychiatric inpatient admission in perinatal period per 1000 births and ethnicity (crude and standardised by age and deprivation) are shown in Table 3. Indian women had statistically significantly lower admission rates than White British women. Black African and Other Black women had higher admission rates; however, this was not statistically significant after standardising for age and deprivation.

**Table 3:** Numbers and rates of inpatient psychiatric admissions per 1000 births by ethnicity (crude and standardised by age and deprivation)

	Ethnicity	All 18+, birth episodes, ***n***	Women with a mental health admission, ***n***	Crude rate (per 1000)			Standardised rate (per 1000)		
				Rate	95% LCL	95% UCL	Rate	LCL	UCL
**A**	**White British**	398,769	443	1.11	1.01	1.22	1.14	1.03	1.25
**B**	**Irish**	3394	**	**	**	**	**	**	**
**C**	**Other White backgrounds**	71,944	67	0.93	0.72	1.19	0.92	0.71	1.18
**D**	**White and Black Caribbean**	3504	7	2.00	0.81	4.12	1.42	0.47	3.30
**E**	**White and Black African**	1629	**	**	**	**	**	**	**
**F**	**White and Asian**	1957	**	**	**	**	**	**	**
**G**	**Other mixed backgrounds**	4459	**	**	**	**	**	**	**
**H**	**Indian**	20,134	9	0.45*	0.21	0.85	0.40*	0.17	0.82
**J**	**Pakistani**	27,052	39	1.45	1.03	1.98	1.32	0.86	1.93
**K**	**Bangladeshi**	9520	19	2.00	1.21	3.12	1.56	0.77	2.82
**L**	**Other Asian backgrounds**	14,653	17	1.16	0.68	1.86	1.01	0.58	1.65
**M**	**Caribbean**	5474	12	2.20	1.14	3.84	1.90	0.88	3.58
**N**	**African**	20,188	39	1.94*	1.38	2.65	1.60	1.05	2.33
**P**	**Any other Black backgrounds**	4630	13	2.82*	1.50	4.81	2.13	1.10	3.73
**R**	**Chinese**	4285	**	**	**	**	**	**	**
**S**	**Other ethnic groups**	23,500	34	1.45	1.01	2.03	1.61	1.05	2.36
	**Total**	615,092	713						

Table shows the number of women with one or more mental health inpatient admissions during the perinatal period (PNP) where the first contact is also within PNP and the total number of birth episodes in 2017 (events with missing ethnicities are included (assumed to be missing at random and are apportioned across all ethnicities in proportion to known ethnicities)). Crude and directly age-standardised rates per 1000 birth episodes are shown along with corresponding 95% confidence intervals

*LCL* lower confidence limit, *UCL* upper confidence limit

*Rate is significantly different (*p* < 0.05) from the White British group

**Numbers are suppressed due to small numbers < 5

The numbers and percentages of women in each ethnic group who were formally detained under the Mental Health Act at their first admission to psychiatric inpatient care are shown in Table 4. White Other women, Asian women (all subgroups), Black African women and Other groups had statistically significantly higher percentages of involuntary admissions than White British women. The numbers of women admitted involuntarily to inpatient care are relatively small, and thus, it was not possible to standardise these rates by age and/or deprivation; however, differences between the key ethnic categories are fairly large.

**Table 4:** Numbers and percentages of women formally detained during the first admission in the perinatal period by ethnicity

	Ethnicity	Women with a mental health admission in the perinatal period, ***n***	Women detained under mental health act during the first admission in the perinatal period, ***n*** (%)	95% LCL (%)	95% UCL (%)
**A**	**White British**	443	130 (29.3)	25.3	33.7
**B**	**Irish**	**	**	**	**
**C**	**Other White backgrounds**	67	38 (56.7)*	44.8	67.9
**D**	**White and Black Caribbean**	**	**	**	**
**E**	**White and Black African**	**	**	**	**
**F**	**White and Asian**	**	**	**	**
**G**	**Other mixed backgrounds**	**	**	**	**
**H**	**Indian**	9	7 (77.8)*	45.3	93.7
**J**	**Pakistani**	39	26 (66.7)*	51.0	79.4
**K**	**Bangladeshi**	19	13 (68.4)*	46.0	84.6
**L**	**Other Asian backgrounds**	17	11 (64.7)*	41.3	82.7
**M**	**Caribbean**	12	4 (33.3)	13.8	60.9
**N**	**African**	39	24 (61.5)*	45.9	75.1
**P**	**Any other Black backgrounds**	13	5 (38.5)	17.7	64.5
**R**	**Chinese**	**	**	**	**
**S**	**Other ethnic groups**	34	17 (50.0)*	34.1	65.9
	**Total**	713	282		

Table shows the number of women aged 18+ who had a birth episode in 2017 who had one or more mental health inpatient admissions recorded during their perinatal period (and where their first recorded contact in the dataset was also within their perinatal period). It provides the number of these who also had a Mental Health Act legal status classification (formally detained under the Mental Health Act) starting on, during or up to 7 days prior to their first admission. The proportion of women with a mental health admission who had a corresponding legal status indicating they were formally detained is shown along with 95% confidence intervals)

*LCL* lower confidence limit, *UCL* upper confidence limit

*Proportion is significantly different (*p* < 0.05) from the White British group

**Numbers are suppressed due to small numbers < 5

### Utilisation of community mental health services

#### Number of attended contacts, non-attendance and cancellations

The numbers and rates of any individual attended community contact with mental health services in the perinatal period are shown in Table 5. All Asian and Black ethnicities as well as White Other, White Irish, Chinese and Other groups had a significantly higher average number of attended contacts during their perinatal period than White British women. When the number of attended contacts is standardised for age and ethnicity, they remain significantly higher for all groups apart from Chinese (and become significantly lower for mixed White/Asian group).

**Table 5:** Numbers of attended contacts within the perinatal period and patients with at least one attended contact during the perinatal period (crude and standardised by age and deprivation)

	Ethnicity	Attended contacts, *n*	Patients, *n*	Crude attended contact rate (per patient)			Age and deprivation-standardised attended contact rate (per patient)		
				Rate	LCL	UCL	Rate	LCL	UCL
A	**White British**	127,291	14,828	8.6	8.5	8.6	8.6	8.6	8.7
B	**Irish**	1459	96	15.2*	14.4	16.0	13.1*	12.3	13.8
C	**Other White**	13,043	1377	9.5*	9.3	9.6	9.3*	9.2	9.5
D	**White/Black Caribbean**	1510	177	8.5	8.1	9.0	9.2	8.6	9.7
E	**White/Black African**	500	57	8.8	8.0	9.5	8.6	7.8	9.5
F	**White/Asian**	434	55	7.9	7.1	8.6	5.0*	4.4	5.7
G	**Other mixed**	1669	185	9.0	8.6	9.5	9.0	8.5	9.5
H	**Indian**	2908	287	10.1*	9.8	10.5	10.1*	9.6	10.7
J	**Pakistani**	5642	487	11.6*	11.3	11.9	11.0*	10.6	11.4
K	**Bangladeshi**	3044	209	14.6*	14.0	15.1	16.8*	16.0	17.5
L	**Other Asian**	3101	292	10.6*	10.2	11.0	9.7*	9.3	10.2
M	**Caribbean**	2599	226	11.5*	11.1	11.9	10.6*	10.2	11.1
N	**African**	5202	396	13.1*	12.8	13.5	12.7*	12.2	13.2
P	**Other Black**	1594	170	9.4*	8.9	9.8	10.1*	9.5	10.8
R	**Chinese**	662	70	9.5*	8.7	10.2	8.3	7.5	9.1
S	**Other**	6116	551	11.1*	10.8	11.4	10.3*	10.0	10.6
	**Not recorded**	1398	370	3.8	3.6	4.0	3.8	3.5	4.0

*LCL* lower confidence limit, *UCL* upper confidence limit

*Proportion is significantly different (*p* < 0.05) from the White British group

Table 6 shows the rates of patient non-attendance/cancellation. Non-attendance/cancellation rates are lower for all Asian groups (including White/Asian group), Black African, White Other, White Irish, Chinese and Other groups and higher for Black Caribbean, Black Other, White/Black Caribbean and White/Black African. When the rates are standardised for age and ethnicity, the rates remain significantly lower for White Other, Pakistani and Black African women and higher for Black Caribbean and White/Black Caribbean women.

**Table 6:** Numbers and rates of non-attendance or patient cancellation per 100 recorded contacts by ethnicity (crude and standardised by age and deprivation)

	Ethnicity	Any individual recorded contact with a mental health team, ***n***	Number of contacts that were not attended or cancelled by the patient, ***n***	Crude DNA rate (per 100 contacts)			Standardised DNA rate (per 100 contacts)		
				Rate	95% LCL	95% UCL	Rate	LCL	UCL
**A**	**White British**	166,947	27,624	16.5	16.4	16.7	16.6	16.4	16.8
**B**	**Irish**	1734	188	10.8*	9.3	12.4	19.5	13.8	25.1
**C**	**Other White backgrounds**	16,308	2238	13.7*	13.2	14.3	14.8*	14.1	15.6
**D**	**White and Black Caribbean**	2041	399	19.5*	17.6	21.5	20.5*	16.9	24.1
**E**	**White and Black African**	693	143	20.6*	17.3	24.0	22.6	14.6	30.5
**F**	**White and Asian**	525	67	12.8*	9.7	15.8	22.3	13.0	31.6
**G**	**Other mixed backgrounds**	2162	370	17.1	15.4	18.9	17.2	14.4	19.9
**H**	**Indian**	3591	493	13.7*	12.5	14.9	16.0	13.3	18.7
**J**	**Pakistani**	7102	897	12.6*	11.8	13.5	12.7*	11.3	14.1
**K**	**Bangladeshi**	3737	519	13.9*	12.7	15.1	14.3	12.0	16.6
**L**	**Other Asian backgrounds**	3774	445	11.8*	10.7	12.9	16.6	13.1	20.0
**M**	**Caribbean**	3438	636	18.5*	17.1	19.9	20.1*	17.6	22.6
**N**	**African**	6481	890	13.7*	12.8	14.6	13.7*	11.6	15.7
**P**	**Any other Black backgrounds**	2171	409	18.8*	17.0	20.7	19.1	16.1	22.1
**R**	**Chinese**	780	82	10.5*	8.2	12.8	21.9	9.8	33.9
**S**	**Other ethnic groups**	7682	1099	14.3*	13.5	15.2	15.4	14.2	16.6
	**Not recorded**	1925	389	20.2*	18.2	22.2	21.9	19.3	24.6
	**Total**	231,091							

*LCL* lower confidence limit, *UCL* upper confidence limit

*Proportion is significantly different (*p* < 0.05) from the White British group

### Density of ethnic minorities and demand or need for mental health care

**Fig. 2:**
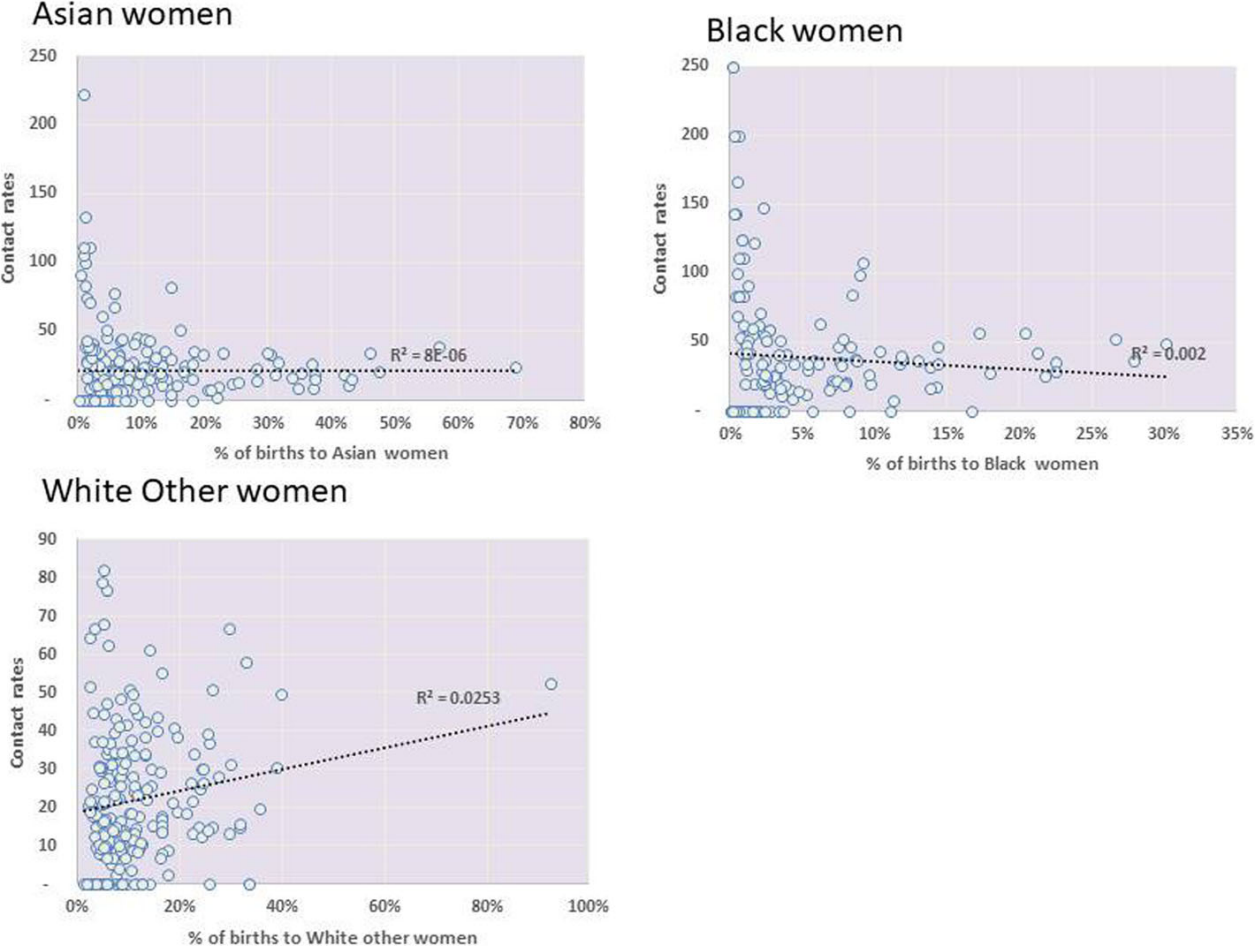
Association between a proxy for ethnic population density and mental health contact rates. Note: shown is the percentage of all birth episodes within individual clinical commissioning groups (CCGs) where mother was of the specified ethnic group (acting as a proxy for the level of ethnic density within an area (CCG)) compared to mental health community contact rates per 1000 birth episodes for that ethnic group. Association between the contact rates is illustrated by linear trend line and coefficient of determination (*R*^2^)

In order to explore whether the higher density of ethnic minorities is associated with lower demand or need for mental health care association between mental health community contact rates and the percentage of births for Black, Asian and White Other groups (as a proxy for population density) within geographical areas related to clinical commissioning groups across England was explored (Fig. 2). No clear association (as indicated on the chart by the coefficient of determination (*R*^2^) value is seen for any of the three ethnic groups considered. Thus, there is no clear evidence that a higher density of ethnic minorities is associated with lower demand or need for mental health care.

## Discussion

### Main findings

During their perinatal period, women from different ethnic groups vary significantly in access to both community mental health services and psychiatric inpatient care. Women in ethnic groups with lower access rates to community mental health services have a higher proportion of involuntary admissions. This applies specifically to Black African, Asian and White Other women compared to White British women. Women from these three groups had a higher number of attended community contacts and fewer non-attendances/cancellations of appointments than White British women indicating that access appears to be a problem rather than engagement.

### Strengths and limitations of the study

To our knowledge, this is the first study that systematically and on a large scale explores access to community and inpatient mental health services for women from different ethnic groups during the perinatal period. The study has several strengths: it uses a near-complete data for a full calendar year of births across England, has high completion rates for ethnicity, examines access to community and inpatient services in the same sample, and provides robust findings that are standardised for age and deprivation and not altered when adjusted for the local density of ethnic minority populations. Although we have relied upon confidence intervals to establish whether there are “statistically significant” differences between the groups, the fact that the study uses a near-complete population means that it can be reasonably argued that any difference can be viewed as significant particularly where the difference is meaningful. The study also has several limitations. For example, the numbers of women admitted involuntarily to inpatient care are relatively small, and thus, it was not possible to standardise these rates by age and/or ethnicity. A further limitation is the assumption that missing ethnicities are missing at random which may not be the case. For example, it is perhaps arguable that missing ethnicities are more likely to be from minority ethnic groups. However, if these were the case, then this would only have the effect of further reducing the contact and admission rates reported within this paper (as a larger proportion of ethnicities are missing from the birth population than from the population that had mental health contact). Additional sensitivity analysis could be carried out to understand the impact on the results of different assumptions about the missing ethnicities*.* However, by using all available data in identified datasets, missing ethnicity data of women with contacts with mental health services were reduced to around 2% and amongst the women with admissions to psychiatric inpatient care to only 0.3%. The study included women aged over 18 due to significant differences in the organisation of child and adolescent mental health services compared to adult services in England as well as different mental health legislation regarding involuntary admissions for subjects under 18. Around 3.7% of births in England were not included in the analysis.

### Comparison with the literature

Previous studies showed separately that people from some ethnic groups access health services less often for common mental disorders [20] and also have higher rates of involuntary admissions to inpatient care [21]. The latter has specifically been shown for Black African and Black Caribbean groups and to a smaller extent for south Asian group [21]. Our study is the first one to demonstrate such differences in community and inpatient care in the same sample on a large scale for women during the perinatal period and specifies the access rates for different groups of ethnic minorities.

That the different access rates simply reflect different levels of need is unlikely. The higher rates of inpatient admissions for the majority of ethnic minority groups and in particular the high proportion of involuntary admissions point towards significant mental health need in these groups and imply that low access to community services is not an indication of low level of need.

Existing literature recommends that amalgamation of ethnic groups should be discouraged to better inform policy and practice [21], and results of our study support this recommendation as access rates vary significantly amongst ethnic categories within large ethnic groups, for example, between White British vs White Other and between Black African vs Black Caribbean.

Primary care and community mental health services should focus on improving equity of access, particularly for Black African, Asian and White Other women. Access to community mental health services for women in their perinatal period could be improved by addressing barriers on different levels, including increasing awareness of perinatal mental illnesses amongst the general population and improving professionals’ expertise in diagnosing and treating perinatal mental health problems. The secondary services should implement the requirements for working with migrants and ethnic minority groups such as organisational flexibility; good interpreting services; closely working with families, social services and community organisations; cultural awareness of staff and making services more culturally relevant; and appropriate information material for all groups [22–24].

For patients from ethnic minorities, pathways to hospital care and rates of involuntary admissions vary substantially. In particular, patients from Black ethnic minority groups were referred to secondary services less often when seen in primary care, had generally more complex pathways to specialist care and were eventually involuntarily admitted to psychiatric inpatient care more often [25]. In our study, only a small proportion of women were involuntarily admitted to a psychiatric hospital, but rates were higher for women from some ethnic groups. Even though absolute numbers of women admitted involuntarily are low, for clinical, ethical, social and economic reasons, treating patients against their will is of particular concern to health care systems and should be prevented as far as possible. Overall, there is limited evidence as to which interventions may reduce involuntary admissions but studies suggest that engagement with community mental health services through crisis planning and self-management interventions can help prevent involuntary admissions [26, 27]. Considering the existing evidence, facilitating the engagement of these groups of women with community services may reduce the risk of involuntary admissions. The current rapid expansion of perinatal mental health services in England offers a unique opportunity to tackle the inequalities identified in this study.

Future research should focus on identifying the precise reasons for the lower access to community mental health services of women from some ethnic minorities during the perinatal period. More detailed data and further research are required about the White Other group. Although this is the largest ethnic minority group in the national record system (10.4% of total births in England in 2017), it is poorly defined [28]. Additional sensitivity analysis could be carried out to understand the impact on the results of different assumptions about the missing ethnicities; this could be informed by work to understand the extent to which missing ethnicity data in routine datasets is skewed to a particular group. Further research should also explore why and how Black Caribbean women engage with community mental health services at a higher rate. As compared to other ethnic groups, a smaller proportion of Black Caribbean women might be the 1st generation migrants [28]. Better engagement with community services may explain the lower rates of involuntary admissions of these women and inform approaches to facilitate engagement with community services. Future studies could perform stratified analyses, i.e. antenatal vs postnatal period to explore the relationship between ethnicity and utilisation of services. Lastly, a unique aspect of the perinatal period that informs mental health service delivery is that antenatal appointments (e.g. with midwife, obstetrician) are often the platform on which to build access to mental health services. Future studies could explore access/utilisation of antenatal care for ethnic minority women.

## Conclusion

Findings from this population-based study in England show that access to mental health services during the perinatal period varies significantly between women from different ethnic groups. Access to community mental health services should be facilitated for Black African, Asian and White Other women during the perinatal period, which may in return reduce rates of involuntary hospital admissions for these groups. The pattern of engagement with community services for women from these ethnicities indicates that access appears to be a problem rather than utilisation.

## Supplementary information

**Additional file 1:.** Supplementary tables.

## Data Availability

The data that support the findings of this study are available from NHS Digital but restrictions apply to the availability of these data, which were used under licence for the current study, and so are not publicly available. Access to data is possible but would be dependent on the recipient agreeing a data sharing agreement with NHS Digital.

## Abbreviations

CCG: Clinical commissioning groups
HRG code: Health Resource Group code
IMD: Index of multiple deprivation
LCL: Lower confidence limit
MHSDS: Mental Health Services Dataset
NCDR: National Commissioning Data Repository
NHS: National Health Service
PNP: Perinatal period
SUS: Secondary uses service
UCL: Upper confidence limit
UK: United Kingdom
US: United States
