# Supplementary material for: Differences in access and utilisation of mental health services in the perinatal period for women from ethnic minorities—a population-based study
Source: BMC Med. 2020 Sep 11;18:245. doi: 10.1186/s12916-020-01711-w (PMC7488566; doi:10.1186/s12916-020-01711-w)
Supplement: Supplementary file 1 — Additional file 1:. Supplementary tables. [file 12916_2020_1711_MOESM1_ESM.docx]

**Supplementary materials**

| **Table 7 - All Births by age group and ethnic group** | | | |  |  |  |  |  |  |  |  |  |  |  |  |  |
| --- | --- | --- | --- | --- | --- | --- | --- | --- | --- | --- | --- | --- | --- | --- | --- | --- |
|  | 1 White | | 2 Mixed | | 3 Asian | | 4 Black | | 5 Other | | Not recorded | | Not Recorded | |  |  |
| Age group | Number of births | % of column | Number of births | % of column | Number of births | % of column | Number of births | % of column | Number of births | % of column | Number of births | % of column | Number of births | % of column |  |  |
| 18-19 | 11305 | 3% | 371 | 4% | 331 | 1% | 369 | 1% | 373 | 2% | 470 | 2% | 1008 | 2% | 14227 | 2% |
| 20-24 | 67727 | 16% | 1630 | 16% | 5814 | 9% | 2926 | 11% | 2665 | 11% | 2916 | 15% | 5675 | 12% | 89353 | 15% |
| 25-29 | 120929 | 29% | 2803 | 27% | 18355 | 29% | 6893 | 26% | 6483 | 26% | 5786 | 29% | 12563 | 27% | 173812 | 28% |
| 30-34 | 131150 | 31% | 3093 | 30% | 23505 | 37% | 8719 | 32% | 8353 | 34% | 6517 | 33% | 15861 | 34% | 197198 | 32% |
| 35-39 | 75072 | 18% | 1917 | 19% | 12735 | 20% | 5998 | 22% | 5456 | 22% | 3550 | 18% | 9159 | 20% | 113887 | 19% |
| 40-44 | 15603 | 4% | 445 | 4% | 2690 | 4% | 1865 | 7% | 1351 | 5% | 695 | 3% | 1978 | 4% | 24627 | 4% |
| 45-49 | 1047 | 0% | 42 | 0% | 206 | 0% | 221 | 1% | 83 | 0% | 53 | 0% | 145 | 0% | 1797 | 0% |
| 50+ | 91 | 0% | 2 | 0% | 19 | 0% | 31 | 0% | 21 | 0% | 9 | 0% | 18 | 0% | 191 | 0% |
| Grand Total | 422924 | 100% | 10303 | 100% | 63655 | 100% | 27022 | 100% | 24785 | 100% | 19996 | 100% | 46407 | 100% | 615092 | 100% |
|  |  |  |  |  |  |  |  |  |  |  |  |  |  |  |  |  |
| **Table 8 - All Births by Index of multiple deprivation index (IMD) and ethnic group** | | | | | | |  |  |  |  |  |  |  |  |  |  |
|  | 1 White | | 2 Mixed | | 3 Asian | | 4 Black | | 5 Other | | Not recorded | | Not Recorded | |  |  |
| IMD decile (1 = most deprived) | Number of births | % of column | Number of births | % of column | Number of births | % of column | Number of births | % of column | Number of births | % of column | Number of births | % of column | Number of births | % of column |  |  |
| 1 | 50721 | 12% | 1802 | 17% | 12983 | 20% | 6313 | 23% | 3967 | 16% | 2665 | 13% | 4394 | 9% | 82845 | 13% |
| 2 | 44731 | 11% | 1626 | 16% | 10652 | 17% | 5889 | 22% | 3513 | 14% | 2815 | 14% | 5353 | 12% | 74579 | 12% |
| 3 | 43282 | 10% | 1281 | 12% | 9287 | 15% | 4273 | 16% | 3391 | 14% | 2386 | 12% | 5392 | 12% | 69292 | 11% |
| 4 | 42321 | 10% | 1048 | 10% | 6717 | 11% | 2728 | 10% | 2657 | 11% | 2032 | 10% | 4892 | 11% | 62395 | 10% |
| 5 | 39769 | 9% | 866 | 8% | 5232 | 8% | 1710 | 6% | 2225 | 9% | 1920 | 10% | 4575 | 10% | 56297 | 9% |
| 6 | 39682 | 9% | 723 | 7% | 3945 | 6% | 1226 | 5% | 1965 | 8% | 1597 | 8% | 4263 | 9% | 53401 | 9% |
| 7 | 37838 | 9% | 682 | 7% | 3046 | 5% | 839 | 3% | 1597 | 6% | 1640 | 8% | 4294 | 9% | 49936 | 8% |
| 8 | 37617 | 9% | 595 | 6% | 2816 | 4% | 701 | 3% | 1420 | 6% | 1511 | 8% | 3910 | 8% | 48570 | 8% |
| 9 | 35686 | 8% | 574 | 6% | 2373 | 4% | 595 | 2% | 1417 | 6% | 1352 | 7% | 3801 | 8% | 45798 | 7% |
| 10 | 31319 | 7% | 485 | 5% | 2120 | 3% | 349 | 1% | 1123 | 5% | 1182 | 6% | 3491 | 8% | 40069 | 7% |
| Not recorded | 19958 | 5% | 621 | 6% | 4484 | 7% | 2399 | 9% | 1510 | 6% | 896 | 4% | 2042 | 4% | 31910 | 5% |
|  | 422924 | 100% | 10303 | 100% | 63655 | 100% | 27022 | 100% | 24785 | 100% | 19996 | 100% | 46407 | 100% | 615092 | 100% |
